# Cafeteria Diet Impacts the Body Weight and Energy Expenditure of Brown Norway Rats in an Apparent Age Dependent Manner, but Has no Effect on Muscle Anabolic Sensitivity to Nutrition

**DOI:** 10.3389/fnut.2021.719612

**Published:** 2021-09-09

**Authors:** Amina El Ayadi, Christian Tapking, Anesh Prasai, Victoria G. Rontoyanni, Doaa R. Abdelrahman, Weihua Cui, Geping Fang, Nisha Bhattarai, Andrew J. Murton

**Affiliations:** ^1^Department of Surgery, School of Medicine, University of Texas Medical Branch, Galveston, TX, United States; ^2^Sealy Center on Aging, University of Texas Medical Branch, Galveston, TX, United States; ^3^Department of Anesthesiology, School of Medicine, University of Texas Medical Branch, Galveston, TX, United States; ^4^Institute of Translation Sciences, University of Texas Medical Branch, Galveston, TX, United States

**Keywords:** obesity, muscle protein turnover, cafeteria diet, high fat diets, energy expenditure, sarcopenia

## Abstract

While obesity blunts the ability of muscle to mount a protein synthetic response to an amino acid infusion in older adults, it is unclear if this insensitivity to nutrition persists long term and in response to complete foods is unknown. To address this, young (2 months old) and old (17–20 months old) Brown Norway rats were randomized to receive either chow or a 12 wk diet of calorie-dense human foods. At wk 10 drinking water was supplemented with 2% heavy water, followed 2 weeks later by a flooding dose of [^2^H_5_]-phenylalanine and an oral leucine bolus, allowing the short and long-term effects of age and diet on muscle protein synthesis rates to be determined. The experimental diet increased energy intake in both young (7.4 ± 0.9%) and old (18.2 ± 1.8%) animals (*P* < 0.01), but only led to significant increases in body weight in the former (young: 10.2 ± 3.0% (*P* < 0.05) and old: 3.1 ± 5.1% (NS) vs. age-matched controls). Notably, energy expenditure in response to the cafeteria diet was increased in old animals only (chow: 5.1 ± 0.4; cafe: 8.2 ± 1.6 kcal.kg b.w^−1^.h^−1^; *P* < 0.05). Gastrocnemius protein fractional synthetic rates in response to either an acute leucine bolus or two weeks of feeding were equivalent across groups irrespective of age or diet. Rodents in old age appear capable of preventing weight gain in response to a calorie-dense diet by increasing energy expenditure while maintaining the anabolic sensitivity of muscle to nutrition; the mechanisms of which could have important implications for the aging obese human.

## Introduction

Obese individuals are at heightened risk of developing frailty in later life, increasing their susceptibility to adverse outcomes including falls, disability, institutionalization, and mortality (1). Recent estimates suggest that more than 30% of the world's population is classified as either being overweight or obese (2), indicating an impending expansion in the size of the overweight elderly population. There is, therefore, a pressing need to understand the causes of frailty in obese populations and develop effective countermeasures that can impede its development.

A key contributor to frailty in later life is the failure to preserve muscle mass, where the ability to synthesize muscle contractile proteins is unable to meet the rate at which they are degraded and subsequently lost from the muscle cell [see (3)]. We and others have shown that the ability of exogenous amino acids to stimulate the synthesis of muscle proteins, a process crucial for the maintenance of muscle protein homeostasis and thereby mass, is blunted with obesity (4, 5). However, the ability of obesity to compromise muscle protein synthetic capacity has not been universally observed. In healthy young obese adults, postprandial muscle protein synthesis rates (6) and mass (7) are typically greater than that seen in age-matched lean counterparts, a likely consequence of excess body weight acting as a mechanical stimulus for muscle growth. However, to date, a direct comparison of the interplay between age and overnutrition on the muscle's capacity to mount an anabolic response to food intake has not been reported. Furthermore, where the impact of obesity on muscle protein metabolism has been examined, it has relied on the use of acute stable isotope methodologies to measure protein fractional synthetic rates in response to exogenous amino acids or proteins over a matter of hours. As such, the chronic impact of overnutrition on habitual muscle protein synthetic capacity remains unknown.

It is permissible that increased adiposity exerts a direct biological effect that compromises the maintenance of muscle mass with obesity. We have shown that the degree of adiposity is inversely related to the anabolic sensitivity of skeletal muscle to exogenous amino acids supply in older men (4). Similarly, utilizing an acute infusion of a lipid emulsion to induce lipid oversupply in lean young men, we have found that the ability of dietary amino acids to stimulate muscle protein synthesis is impaired (8). Collectively, these findings implicate increased availability of lipids as a potential causative feature for the obesity-induced impairment of muscle anabolic sensitivity to amino acids. Given that muscle mitochondrial respiratory function is purportedly diminished with old age (9), reduced muscle oxidative capacity coupled with increased lipid availability may act in a synergistic fashion to exacerbate muscle anabolic resistance to exogenous amino acids.

In the current report we examine the dual impact of age and exposure to a prolonged high-fat, high-calorie diet on chronic and acute measures of muscle protein synthesis in sedentary rodents, and its association with impaired mitochondrial bioenergetics. We hypothesized that older animals will display hallmarks of impaired mitochondrial function, leading to the prolonged blunting of muscle protein synthesis in response to nutrition.

## Methods

### Animals

Sixteen 17- to 20-month-old male and female Brown Norway rats were obtained from the Aged Rodent Colony at the National Institute on Aging via Charles River Laboratories, in addition to 16 young (2 months old) male and female animals of the same strain and from the same commercial supplier. Animals were pair housed with a sex and age-matched cage-mate and kept under a 12-h light/dark cycle in temperature-controlled vivarium facilities located at the University of Texas Medical Branch. The cages utilized in the study lacked a running wheel, preventing the animals from performing voluntary exercise. Animals were allowed to acclimatize to their new surroundings for a minimum of one week before the commencement of experimental procedures. The protocol was approved by the University of Texas Medical Branch's Institutional Animal Care and Use Committee (Ref #: 1810078).

### Cafeteria Diet

After the required acclimatization period, animal cages were randomized to receive either an experimental diet designed to induce hyperphagia, termed cafeteria diet (CAFE), or standard laboratory chow (Rodent Diet 20, PicoLab; CON), with diets maintained for 12 weeks. While various competing models of diet-induced obesity exist, the cafeteria model has proven to be a translatable model reflective of human obesity, where 12 weeks provision of the experimental diet is associated with sustained weight gain, and the development of hallmarks associated with metabolic syndrome (10, 11).

Animals assigned to CAFE were provided daily with four energy-dense, highly palatable human foods out of a selection of nine items total. The items provided were beef jerky, candy, caramel-coated popcorn, chocolate, cookies, donuts, honey-roasted peanuts, potato chips, and processed cheese; the nutritional information of the provided foods can be found in Table 1. Food items were provided to animals in excess and each day one of the four food items was swapped to maintain novelty and encourage hyperphagia, an approach that has previously proven successful (11). Throughout the 12-week period, animals retained unrestricted access to standard chow and water. Experimental food items and chow were weighed on a daily basis to allow food intake to be estimated, with the measured mass of food items adjusted to account for daily evaporative loses (Table 1). During the course of the study, one of the old animals assigned to the chow diet showed signs of rapid deterioration and was euthanized prematurely; their data were excluded from the study.

**Table 1:** Nutritional information of experimental diet.

**Composition per gram**	**Peperoni**	**Candy**	**Caramel-coated popcorn**	**Chocolate**	**Cookies**	**Donuts**	**Honey-roasted peanuts**	**Potato chips**	**Processed cheese**	**Standard chow**
Calories (kcal)	4.3	3.3	5.2	5.3	5.0	4.3	5.7	5.7	2.9	3.5
Total fat (g)	0.37	0.02	0.23	0.30	0.29	0.23	0.46	0.39	0.21	0.05
Saturated fat (g)	0.13	0.00	0.06	0.17	0.21	0.12	0.07	0.05	0.11	0.01
Cholesterol (mg)	0.83	0.00	0.16	0.33	0.00	0.17	0.00	0.00	0.71	0.001
Total carbohydrates (g)	0.07	0.78	0.71	0.60	0.61	0.53	0.25	0.54	0.04	0.39
Total sugars (g)	0.03	0.48	0.39	0.53	0.36	0.28	0.14	0.04	0.04	0.05
Protein (g)	0.20	0.04	0.06	0.07	0.04	0.03	0.25	0.04	0.21	0.20
Sodium (mg)	16.7	2.22	3.23	0.67	1.96	2.67	3.75	4.82	7.14	3.0
Evaporative loss (%/day)	9.6	0	9.6	−0.8	−2.6	2.6	1.1	−2.6	15.0	-

*With the exception of evaporative losses which was determined experimentally, the reported values are as stated by the respective manufacturer and expressed per gram*.

### Energy Expenditure

At week 8, animals were individually housed for 48 h in cages connected to an indirect open circuit calorimeter (Comprehensive Lab Animal Monitoring System, Columbus Instruments), to allow the determination of energy expenditure. The incorporated exercise wheels of the metabolic cages were locked in position to prevent the animals from performing exercise while housed in the cages. Animals were provided with their standard chow and/or experimental food throughout the two-day period, but experimental foods were not weighed or rotated during the period to avoid unnecessarily disturbing the animals. The first 24 h of data from the period of recording was discarded to allow the animals time to adjust to the novel environment. On occasion, the indirect calorimeter was found to be operating outside the equipment's accepted tolerances when calibrated against known references gases. Data from affected runs were subsequently discarded, resulting in data derived from several animals being removed. At the end of the study, complete data from 20 animals remained (young: 6 animals/group; old: 4 animals/group).

### Determination of Muscle Protein Synthesis Rates

To allow the effect of age and cafeteria diet on habitual rates of muscle protein synthesis to be determined, the incorporation of deuterated alanine into mixed muscle proteins was measured over a two-week period using a previously described heavy water labelling approach (12, 13). In short, the approach relies on the known ability of intracellular alanine to become deuterated via transamination reactions when body water is enriched with deuterium, occurring in a consistent and predictable manner (14). Ten weeks after the start of the dietary intervention period, all animals received a 10 ml/kg i.p. bolus of 70% atom percent excess (APE) heavy water (^2^H_2_O) to prime the body water pool. Afterwards, cage drinking water was supplemented with 2% ^v^/_v_ of the same heavy water and maintained for the remainder of the study.

To establish the impact of age and cafeteria diet on the muscle's ability to mount a protein synthetic response to an acute anabolic stimulus, a flooding dose of [^2^H_5_]-phenylalanine was administered to the same animals while under conditions of hyperinsulinemia and hyperleucinemia. Isotopically labelled phenylalanine was selected as a tracer for protein synthesis on the basis that it is neither synthesized nor oxidized by skeletal muscle. Twelve weeks after the start of the experiment, the animals underwent an overnight fast. The following morning, all animals received an i.p. injection of 0.5 U/kg insulin in combination with a 0.34 g/kg b.w. oral gavage of leucine, dissolved in phosphate buffered saline (PBS). It has been previous shown that leucine is the principle amino acid responsible for the stimulation of muscle protein synthesis following the exogenous administration of protein and/or essential amino acids (15). Thus, the use of leucine here mimics the response expected following the consumption of a meal without diluting circulating [^2^H_5_]-phenylalanine enrichment. Furthermore, a 0.34 g/kg b.w. oral bolus of leucine has been shown to stimulate muscle protein synthesis, with a maximal response reported following a 1.35 g/k.g. b.w. bolus (16). A submaximal dose was selected to avoid masking the presence of muscle anabolic resistance to amino acids as could occur with a large bolus (17). Thirty minutes after the administration of leucine/insulin, a 50 mg/kg b.w. bolus of [^2^H_5_]-phenylalanine, dissolved in PBS, was administered via the tail vein of the animal. After a further 30 min, animals were sacrificed by decapitation, with the gastrocnemius muscle rapidly dissected and frozen in liquid nitrogen prior to analysis. The gastrocnemius muscle was chosen based on its mixed fiber type resembling the typical composition seen in the human vastus lateralis (18), a muscle commonly studied in human metabolic trials.

### Tissue Processing and Analysis

Collected muscle samples were processed for the determination of [^2^H_5_]-phenylalanine and deuterated-alanine incorporation in muscle proteins as described previously (12, 13). Briefly, approximately 30 mg of muscle tissue was homogenized in 10% perchloric acid before being centrifuged at 3,000 rpm for 10 min at 4°C. Afterwards, the supernatant containing the free intracellular amino acids were collected. The pellet containing the mixed muscle proteins was washed in 2% perchloric acid, centrifuged at 3,000 rpm for 10 min at 4°C, and the supernatant discarded. The resultant pellet was washed two times in 100% ethanol and once in ethyl ether before being centrifuged at 3,000 rpm and the supernatant discarded as before. Afterwards, tubes containing the pelleted proteins were dried overnight at 50°C, before being hydrolyzed for 24 h in 6 N HCl at 110°C. On completion, the hydrolyzed muscle proteins and previously collected supernatant were purified by passing through a chromatography column containing a cation resin (AG 50 W 200–400 mesh, Bio-Rad) charged with 1 N HCl. After repeated cycles of washing with ddH_2_O, amino acids were eluted from the columns with 2 N NH_4_OH before drying overnight in an evaporator (SpeedVac, Thermo Fisher Scientific). Dried samples were treated with 80 μl of acetonitrile:N-tert-butyldimethylsilyl-N-methyltrifluoroacetamide (1:1) and heated at 95°C for 40 min to produce tert-butyldimethylsilyl derivatives of the amino acids. The abundance of [^2^H_5_]-phenylalanine in processed samples was measured on a gas chromatography-mass spectrometer (6890N GC coupled to a 5973N mass spectrometer, Agilent Technologies), in parallel with [^2^H_5_]-phenylalanine standard curves encompassing the range of enrichments expected in samples. For intracellular free amino acids, ions m/z 336 and 341 were monitored, while for bound protein, ions m/z 237 and 239 were examined. The enrichment of samples with deuterated alanine were examined on a gas-chromatography-pyrolysis-isotope ratio mass spectrometer (Trace 1310 GC coupled to a Delta V IRMS via a high-temperature thermal conversion oven, Thermo Fisher Scientific), respectively, as described previously (12, 13, 19).

### Calculations

Irrespective of the isotope method employed, the fractional synthetic rate (FSR) of mixed muscle proteins was calculated using the standard precursor-product method as described below, where *E*_*m*1_ and *E*_*m*2_ represent the enrichment of mixed muscle proteins by the labelled amino acids at baseline and time of sacrifice, respectively.


(1) $$\begin{array}{l} \text{FSR }\left(\text{\%}.\text{da}{\text{y}}^{-1}\right)=\left(\frac{E_{m2}-\;E_{m1}}{E_{precursor}\;x\;t}\right)\times \;100 \end{array}$$

Given that it was not feasible to collect muscle from the animals prior to sacrifice, for deuterated alanine, baseline enrichment was calculated based on the expected natural deuterium abundance within the amino acid (0.015507 %). In the case of phenylalanine, natural incorporation of isotopes leading to a m+5 mass shift was considered negligible, and E_m1_ was considered zero. *E*_*precursor*_ was taken as the enrichment of the intracellular free amino acid fraction at time of sacrifice in both cases. *t* represents the length of time of the isoptope administration protocol, which was 14 and 1/24 days for heavy water and D_5_-phenylalanine, respectively.

### Assessment of Muscle and Brown Adipose Tissue Mitochondrial Function

Muscle mitochondrial function was assessed by high-resolution respirometry in gastrocnemius muscle as previously described (9, 20). The gastrocnemius, as described above, was selected based on the muscle closely resembling the mixed fiber type of human vastus lateralis. Sample constraints of the high-resolution respirometer prevented other rodent hindlimb muscles, such as the tibialis anterior (fast-twitch) and soleus (slow-twitch), from being examined in parallel.

Approximately 5 mg samples collected from the mid-belly of the muscle at time of dissection were placed in ice-cold preservation buffer (BIOPS; pH 7.1) consisting of 5.77 mM sodium ATP, 2.77 mM CaK_2_-EGTA, 15 mM sodium phosphocreatine, 0.5 mM dithiothreitol, 20 mM imidazole, 7.23 K_2_-EGTA, 50 mM MES hydrate, 20 mM taurine and 6.56 mM MgCl_2_, before same-day analysis. Muscle samples were dissected in fresh BIOPS solution using sharp forceps to reveal myofiber bundles. Afterwards, the sarcolemmal membrane of the myofiber bundles was permeabilized by incubation for 10–15 min at 4°C in BIOPS buffer containing 50 μg/ml saponin. Afterwards, myofiber bundles were washed with fresh BIOPS buffer to remove remaining saponin, before being weighed and transferred into the chamber of an Oxygraph-2k high-resolution respirometer (Oroboros Instruments) containing 2 ml of MIRO5 buffer (0.5 mM EGTA, 20 mM HEPES, 60 mM lactobionic acid, 10 mM KH_2_PO_4_, 3 mM MgCl_2_, 110 mM sucrose, 20 mM taurine and 1 mg/ml essential fatty-acid free bovine serum albumin; pH 7.1).

Mitochondrial respiration of permeabilized myofiber bundles was determined via the sequential titration of tricarboxylic acid (TCA) cycle substrates (5 mM pyruvate, 1.5 mM octanoyl-l-carnitine 2mM malate and 10 mM glutamate) to induce state 2 (leak) respiration; 5 mM ADP to induce state 3_i_ respiration); 10 mM succinate to induce state 3_i+ii_ respiration and thereby maximal phosphorylating respiration; 10 μM cytochrome c and 5 μM carbonyl cyanide m-chlorophenyl hydrazone to induce maximal respiration (maximal electron transfer capacity). Collected respiratory trace data was examined for the identification of steady state conditions following the addition of each compound and used to calculate respiratory flux data as described previously (9).

Given the unique role of brown adipose tissue (BAT) in energy metabolism, coupled with previous studies demonstrating that activity of the tissue is modulated by the provision of high-fat diets (21, 22), samples of BAT were collected at time of euthanasia and their respiration examined by high-resolution respirometry using a protocol described previously (23). On collection, tissue samples were stored in fresh BIOPS solution, before being analyzed later the same day. When required, 1–3 mg of BAT was transferred to a chamber of the respirometer containing MIRO5, with the samples permeabilized by the addition of 2 μM digitonin. Afterwards, uncoupled Adenosine triphosphate (ATP) production was examined by the addition of the substrates glutamate (10 mM), malate (2 mM), octanoyl-l-carnitine (1.5 mM), pyruvate (5 mM) and the oxygen concentration of the chamber examined. On completion, uncoupled protein-1 (UCP-1) dependent uncoupled respiration was determined through the titration of guanosine-5′-diphosphate (GDP), a potent inhibitor of UCP-1.

### Statistics

To account for differences in body weight between male and female animals, nutrient intake and energy expenditure were expressed relative to body weight. For similar reasons, body weight was expressed as percentage change from baseline rather than absolute values. Differences between groups were identified through use of a two-way ANOVA, with a Fisher's LSD test used to locate significant differences when an interaction between main terms was found to be significant. Based on the above statistical approach and the stated hypothesis, to detect a significant interaction between factors (age × diet) when examining acute measures of muscle protein fractional synthetic rate (the primary endpoint), would require a sample size of 24 animals (6 per group) achieving >80% power with a type I error rate of 0.05. This is based on the publication by Crozier and colleagues (16), demonstrating a 30% (±11% SD) increase in muscle protein fractional synthetic rate with a 0.34 g oral leucine bolus and assuming equal variances between groups. In the case of energy expenditure, values during the light and dark cycle were averaged, and the difference examined by two-way ANOVA as above. All statistical tests were performed using the Prism statistical software (v8.4.3; GraphPad), with significance accepted at P < 0.05. Unless otherwise stated, all data is reported as means ± SEM.

## Results

**Figure 1:**
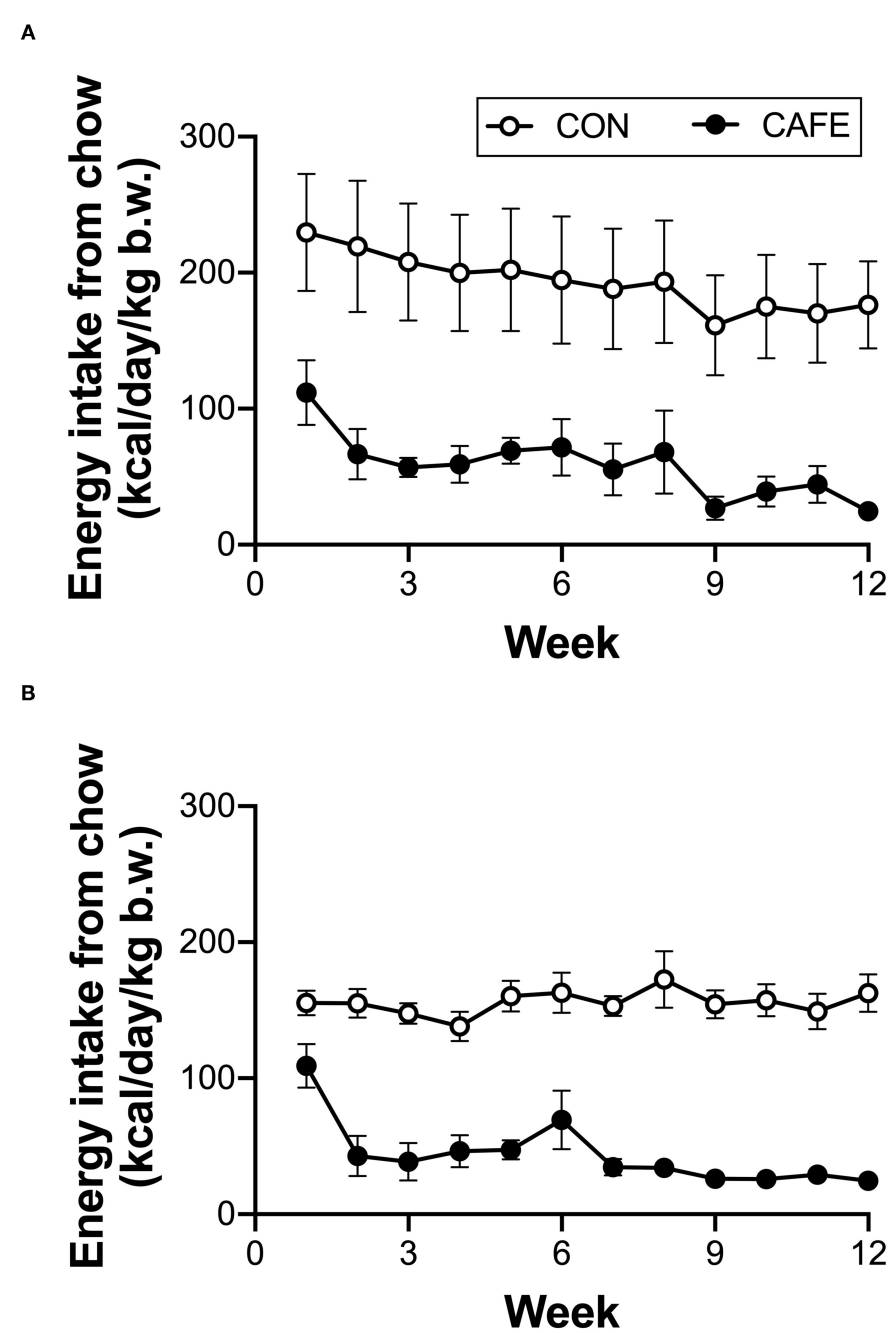
Longitudinal impact of experimental cafeteria diet on chow consumption in young **(A)** and old **(B)** Brown Norway rats. A significant effect of the cafeteria diet on chow consumption was observed in both young (*P* < 0.05) and old (*P* < 0.001) animals. Values represent cage averages ±SEM. *n* = 4 cages/group.

Across the 12-week intervention period, there was no significant difference in the energy or macronutrient intake between young and aged animals on a per body weight basis when the animal's sole food source was standard laboratory chow (Table 2). The provision of highly palatable energy-dense human foods resulted in a 77 ± 2 and 75 ± 2% decline in chow consumption over the 12-week period in both young and old animals, respectively (*P* < 0.001; Figure 1). Despite a decline in chow consumption with the experimental diet, overall energy intake across the 12-week period was increased by 7.4 ± 0.9% (range: 5.2–9.7%) in young, and 18.2 ± 1.8% (range: 17.1–22.9%) in aged animals when compared to sex and age matched controls (*P* < 0.01; data not shown). While absolute energy intake increased with the cafeteria diet, when normalized to body weight, calorie consumption was equivalent between groups irrespective of diet or age status (Table 2).

**Table 2:** Daily nutrient intake and gastrocnemius muscle weights.

	**Young**		**Aged**	
	**CON**	**CAFE**	**CON**	**CAFE**
Energy intake (kcal/kg b.w.)	193 ± 41.5	244 ± 41.4	156 ± 9.7	177 ± 11.2
Total fat (grams/kg b.w.)	2.7 ± 0.6	11.8 ± 1.9^***^	2.2 ± 0.14	8.2 ± 0.6^**^
Saturated fat (grams/kg b.w.)	0.51 ± 0.11	5.1 ± 0.8^***^	0.41 ± 0.03	3.4 ± 0.2^***^, ^†††^
Cholesterol (mg/kg b.w.)	0.08 ± 0.02	13.6 ± 2.6^***^	0.06 ± 0.00	8.4 ± 1.1^**^
Total carbohydrates (grams/kg b.w.)	21.3 ± 4.6	24.5 ± 4.1	17.2 ± 1.0	18.6 ± 1.3
Sugar (grams/kg b.w.)	2.7 ± 0.6	12.7 ± 2.0^***^	2.2 ± 0.14	9.7 ± 1.0^***^
Protein (grams/kg b.w.)^‡^	10.9 ± 2.3	7.7 ± 1.4	8.8 ± 0.6	5.3 ± 0.3
Gastrocnemius muscle weight (mg)	M: 2022 ± 88 F: 1034 ± 66	M: 1425 ± 130F: 1229 ± 113	M: 1494 ± 137 F: 947 ± 58	M: 1347 ± 275F: 1056 ± 101

*N = 4 cages/group. CON, Control diet; CAFÉ, high-fat calorie-dense diet; M, Male; F, Female. Excepted where stated, values expressed per kilogram of body weight and represent means ± SEM*.

** *P < 0.01*,

*** *P < 0.001: significantly different from aged-matched control*.

††† *P < 0.001: significantly different from young cafeteria treated animals*.

‡ *P < 0.05: significant main effect of cafeteria diet*.

The dietary intervention employed was highly effective at modulating the animal's macronutrient intake (Table 2). The CAFE treated young and aged animals consumed 4.4× and 3.7× greater amounts of total fat compared to their chow consuming young and aged counterparts, respectively (*P* < 0.01). Likewise, increased intake of cholesterol and saturated fat was also observed with the CAFE diet, although the latter was significantly less pronounced in the aged animals (increased 10× vs. 8.3× in young and old animals, respectively). While total carbohydrate intake was unaffected by either age or the dietary intervention, the CAFE diet quadrupled added sugar intake in both age groups (*P* < 0.001). In contrast to the increased fat and sugar intake seen in CAFE treated animals, a comparable fall in protein intake was observed in response to the experimental diet in young and aged animals, respectively (main effect: *P* < 0.05).

**Figure 2:**
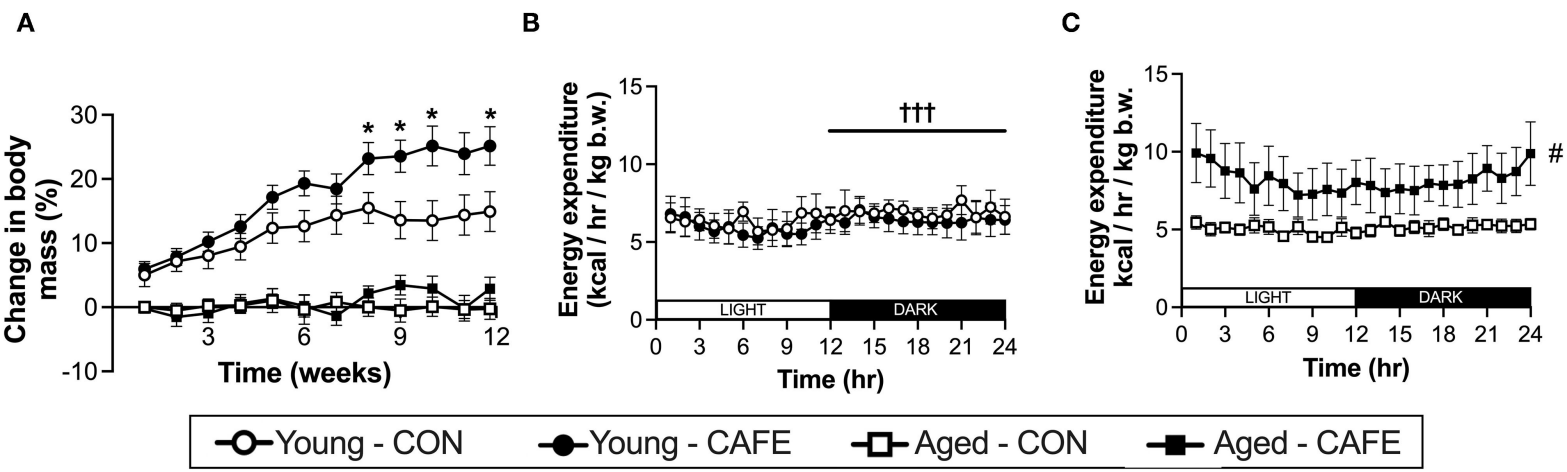
Impact of an experimental cafeteria diet on body weight and energy expenditure in young and old Brown Norway rats. Changes in body mass reported relative to baseline in young and old animals across the 12-week intervention **(A)**. Energy expenditure examined by indirect calorimetry after 8 weeks of the dietary intervention, expressed per kg of body weight **(B,C)**. *P < 0.05: significantly different from time and age-matched control. ^#^*P* < 0.05: significant main effect of dietary intervention. †††*P* < 0.001: significantly different from light phase. Values represent means ± SEM. *n* = 4–8/group.

While CAFE treated animals increased their absolute energy intake in response to the experimental diet, its impact on body weight differed dependent on age, with a significant interaction between the impact of the dietary intervention and time on body mass in young (P < 0.001), but not old animals (Figure 2A). Throughout the 12-week period of examination, young animals gained weight irrespective of diet (main effect of time: *P* < 0.001), however from week 8 onwards, the body mass of CAFE treated animals was greater than that of their chow treated counterparts (*P* < 0.05). In sharp contrast, the body mass of aged animals was static throughout the 12-week period, regardless of the diet provided. Neither cafeteria diet nor age had a detectable effect on gastrocnemius muscle weight (Table 2), a potential consequence of the small animal numbers per group (*n* = 3 to 4/group) when animals were separated by sex—a requirement given the weight differences seen between male and female animals.

To understand the discord between energy intake and body weight with age, 8 weeks following commencement of the experimental diet, the energy expenditure of the animals was examined by indirect calorimetry. Utilizing this approach, energy expenditure normalized to body weight, was increased with the CAFE diet in aged (*P* < 0.05) but not young animals (Figures 2B,C, respectively). In contrast, energy expenditure was greater in the dark cycle (hours 12–24), compared to light cycle in the young animals only (*P* < 0.05), a period when rodents are typically more active (24). No discernable difference in the response to the time of day was observed between diets.

**Figure 3:**
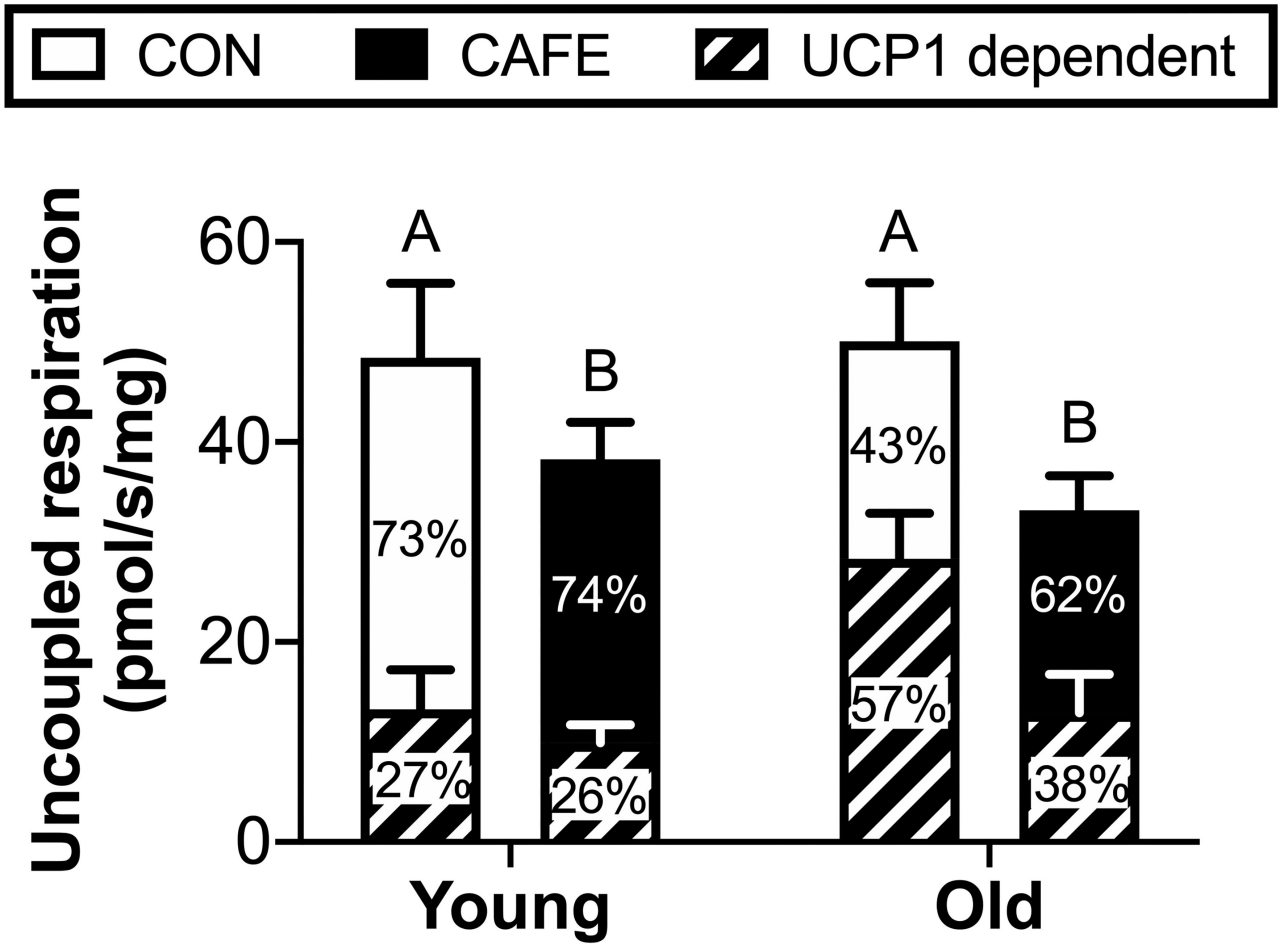
Uncoupled mitochondrial respiration rates of brown adipose tissue in Brown Norway rats subjected to a 12-week cafeteria diet. Main bars represent total uncoupled respiration normalized to tissue weight. Shaded and non-shaded areas of each bar represent the contribution of UCP1-dependent and UCP1-independent respiration to total uncoupled respiration, respectively. Values represent means ± SEM. Bars with differing letters denote significance (*P* < 0.05). *n* = 6–7/group.

To ascertain potential sources of the increased energy expenditure seen in CAFE treated aged animals, the respiratory function of gastrocnemius muscle and brown fat were assessed by high resolution respirometry at week 12. Neither diet nor age modified leak, phosphorylating state 3, or maximal mitochondrial respiratory capacity of skeletal muscle mitochondria (Table 3). Similarly, no significant differences in respiratory flux control ratios, a function of the above, were observed between groups (data not shown). In contrast, the uncoupled respiratory capacity of brown fat, a thermogenic tissue, was suppressed by the CAFE diet to equivalent degrees in young and old animals (Figure 3; *P* < 0.05). Notably, the proportion of UCP1 mediated uncoupled respiration was static in young animals irrespective of treatment; in old animals UCP1 dependent respiration represented a greater proportion of total uncoupled respiration (*P* < 0.01). Despite old animals subjected to the CAFE diet showing greater numerical rates of UCP1 dependent uncoupled respiration than that of their chow treated counterparts, no significant interaction between age and diet was observed.

**Table 3:** Mitochondrial respiratory capacity in gastrocnemius permeabilized myofibers in response to experimental cafeteria diet. Determined following 12-week exposure to either a control (CON) or high-fat calorie dense diet (CAFE).

**Mitochondrial respiration (pmol/sec/mg)**	**Young**		**Aged**	
	**CON**	**CAFE**	**CON**	**CAFE**
Leak—State 2	2.85 ± 0.69	3.54 ± 0.75	2.19 ± 0.28	3.10 ± 0.65
Phosphorylating—State 3_i_	62.9 ± 12.7	64.1 ± 12.7	55.8 ± 8.7	70.8 ± 7.0
Phosphorylating—State 3_i+II_	65.2 ± 14.6	67.4 ± 14.0	59.3 ± 8.9	73.2 ± 7.1
Maximal respiration (electron transfer capacity)	76.8 ± 17.0	82.2 ± 17.4	73.1 ± 11.1	84.4 ± 6.9

*Values represent means ± SEM. No significant differences observed between groups. n = 7–8/group*.

**Figure 4:**
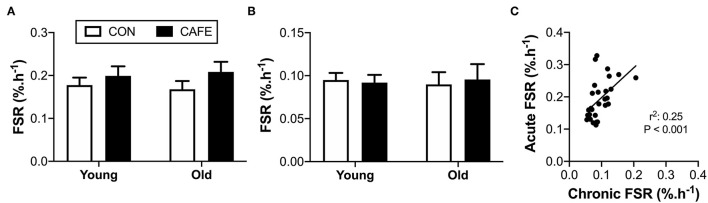
Effect of 12-week cafeteria diet on mixed-muscle protein fractional synthetic rate in young and old Brown Norway rats. Mixed-muscle protein fractional synthetic rate assessed **(A)** acutely following an oral leucine bolus, performed under hyperinsulinemic conditions, and **(B)** chronically, performed over a two-week period. Values represent means ±SEM. No significant differences observed between groups. **(C)** Relationship between acute and chronic measures of mixed muscle protein synthetic rate, performed using two alternative stable-isotope labelling methodologies. Significant correlation between acute and chronic measures of mixed muscle protein fractional synthetic rate observed (*P* < 0.001). *n* = 6–8/group.

While diet and age exerted effects on energy intake, body weight, and energy expenditure in the examined animals, there appeared little effect of the diet on mixed muscle protein fractional synthetic rates in response to either variable. Muscle protein fractional synthesis rates were equivalent across groups irrespective of age or diet, both when the response to an acute leucine bolus (Figure 4A) or two weeks of feeding (Figure 4B) were independently considered. Given the inability to detect significant differences in muscle protein synthesis due to the interventions, the sensitivity of the isotope-labelling techniques to detect biologically meaningful changes in muscle protein synthesis rates were confirmed by examining the correlation between the two methods (Figure 4C). A significant (*P* < 0.001) linear relationship was observed between the two competing approaches, demonstrating that both techniques had the ability to detect an animal's innate protein synthetic capacity in response to anabolic stimuli (i.e., the techniques could detect high and low responders). As such, both methods appeared to possess the sensitivity required to detect biologically meaningful differences between groups, but no such differences existed.

## Discussion

The incidence of obesity in western elderly populations is continuing to increase (2), yet the impact of overnutrition on muscle metabolism is poorly understood despite the tissue's central role in avoiding frailty and maintaining overall good health. Utilizing an established rodent model of diet induced obesity that is considered to reflect human pathological changes to excess energy intake (10, 11), we demonstrate that overconsumption of a calorie-dense, high-fat diet does not impair muscle protein synthesis over both the short (minutes) and long-term (weeks). We also demonstrate that the impact of CAFE diet on body weight differs between young and old animals, with the older animals able to effectively stave off weight gain by increasing energy expenditure. While the mechanisms by which this occurs in rodents remains elusive, if identified it could have significant implications in the quest to develop effective weight gain prevention treatments for aging populations.

The apparent discord between weight gain and age with the cafeteria diet has previously been noted by Teixeira and colleagues (25). Like the current results, they found that significant weight gain was observed in young (3 month) but not old (18 month) Wistar rats. However, this appeared a reflection of the experimental diet's ability to encourage hyperphagia only in younger animals, with energy intake unaltered between chow and cafeteria diet treated older animals. In sharp contrast, Warneke and colleagues, comparing the impact of cafeteria diet between juvenile (6 weeks) and adult (12 month) Sprague-Dawley rats observed weight gain in the older animals only, occurring within a week following the onset of the experimental diet (26). Reasons for the conflicting observations between studies are not readily apparent, but it is notable that the strain of rat examined differed across all three studies. The Brown Norway rat was selected in the current study based on its established use in the study of human aging related pathologies. Another defining feature between studies is the age of animals examined, with our study utilizing animals in the later stages of their lifespan compared to the other reports. Regardless of the underlying discord between studies, our observations that the Brown Norway rat is able to increase energy expenditure in old age to offset increased calorie intake is noteworthy and has significant ramifications for the treatment of obesity in elders. Given that the animals had no access to exercise apparatus such as running wheels, and that energy expenditure was consistently increased in both the light and dark phase, it is unlikely that changes in physical activity explain the observed differences in energy expenditure. Furthermore, while the mitochondrial function of key metabolic tissues was determined in an attempt to establish the source of the increased energy expenditure in older cafeteria fed animals, it was apparent that this was not due to changes in mitochondrial respiratory capacity in either muscle or brown adipose tissue.

Somewhat unexpectedly, the cafeteria diet was found to suppress mitochondrial uncoupling in brown adipose tissue, occurring in both young and old animals to equivalent degrees. Previous research examining the impact of high fat diets in rodents had observed contrary findings, with thermogenesis increased concomitant with enhanced brown fat mitochondrial respiration (27). One possible explanation for the discrepancy between studies is our decision to fast animals prior to acute metabolic assessment. This was performed to promote the stimulation of muscle protein synthesis in response to a leucine oral gavage and eliminate the effect of recent food consumption on muscle protein synthesis between animals. However, it has been demonstrated that the fasting of rats previously subjected to an obesogenic diet results in the increased inactivation of uncoupling proteins in brown adipose tissue mitochondria compared to that of their chow-treated counterparts (28). Whether this occurred in the current study is unknown.

In 2013, Cypess et al. reported that adult humans possess functional BAT, as determined by 18F-fluorodeoxyglucose positron-emission tomography (PET) computed tomography (CT) scanning, with BAT obtained by biopsy staining positive for UCP-1 (29). Moreover, the same authors demonstrated that the presence of functional BAT was inversely related to both age and body mass index. While in the current study no effect of age on BAT mitochondrial uncoupling was observed, our results suggest that diet may contribute to the decline in BAT function seen with obesity in humans, providing novel avenues for investigation.

It has previously been observed that ageing decreases coupled mitochondrial respiration in human skeletal muscle (9). Similarly, we have observed that the transcription of genes associated with mitochondrial function (cytochrome-c, PPARα, PGC-1α and transcriptional factor A mitochondrial) are decreased in the muscle of older obese adults compared to their lean counterparts (4), thereby suggesting that age and obesity may act as synergistic factors impairing muscle mitochondrial function. In contrast to observations pertaining from human volunteers, in the current study we did not observe any effect of age or obesity on muscle mitochondrial function. Irrespective of whether leak, phosphorylation, or maximal mitochondrial respiratory capacity was being considered, the impact of diet or age failed to modulate respiratory function. Given the argument that muscle mitochondrial function is a reflection of deconditioning in old age, where activity status affects mitochondrial respiratory function, it is permissible that the lack of exercise apparatus resulted in comparable declines in mitochondrial function in all groups and masked any effect of age or diet. Nonetheless, our results demonstrate that neither age nor consumption of calorie-dense high-fat foods in rodents culminates in the committed deterioration of muscle mitochondrial function.

Previously, we and others, have demonstrated that the synthetic capacity of skeletal muscle to respond to the acute administration of either amino acids or protein isolate is diminished in the older obese adult (4, 30). Likewise, others have shown that the myofibrillar protein synthetic response to animal protein (pork), is blunted in both overweight and obese young adults, unlike their health weight counterparts (5). However, this has not been universally observed, with reports of obesity having no effect on either fasting or postprandial myofibrillar protein synthesis rates (31). Notably, Smeuninx and colleagues observed that the protein synthetic response to milk protein isolate is positively correlated with average daily step-count in older adults (30), suggesting that the activity status of studied participants could have a bearing on the magnitude of muscle anabolic resistance to amino acids observed. However, given observations by the same researchers that a negative association exists between the abundance of leg fat and the ability of muscle to mount a protein synthetic response to protein, it is not currently possible to dissociate the causative effects, if any, of sedentary behavior and intramuscular lipid accumulation on muscle protein metabolism.

Whether a resistance to dietary protein occurs over prolonged periods, where individuals are consuming their typical diet is unknown. To address this, we examined the acute protein synthetic response to a leucine challenge following the high-calorie high-fat dietary challenge, while simultaneously determining the chronic effects of the experimental diet on muscle protein synthesis, examined over a two-week period in both young and old rodents. Utilizing this approach, we observed that neither age nor consumption of calorie-dense, high-fat human foods impaired the protein synthetic response to leucine (acute) or food (chronic) in the Brown Norway rat. The inability to detect changes in muscle protein synthesis using the experimental techniques employed in the current study did not appear due to their inability to detect physiologically meaningful changes in protein synthetic rates, as highlighted by the techniques ability to identify high and low responders to anabolic cues.

While we are the first to report the impact of an obesogenic diet on chronic muscle protein synthesis rates, it is notable that others have also observed an inability of diet-induced obesity to induce muscle anabolic resistance acutely in rodents. De Sousa and colleagues observed that diet induced obesity, irrespective of whether weight gain was due to a high fat diet, sucrose, or cafeteria diet, failed to modulate muscle protein synthesis as determined by 30 min puromycin incorporation (32). Notably, in the aforementioned study, muscle protein synthesis rates were unchanged despite significant increases in gastrocnemius lipid accumulation with high fat feeding and cafeteria diet, suggesting that the intramuscular accumulation of lipid with obesity in rodents does not preclude the tissue from being able to mount a satisfactory anabolic response to dietary amino acids. Masgrau et al. investigating the time course of muscle anabolic resistance with high fat feeding in rats, observed that declines in muscle protein fractional synthesis rate are only evident after long-term (24 week) high fat feeding, and are not seen with short-term (16 week) provision of the same diet (33). As with other published reports, Masgaru and colleagues only examined muscle protein synthesis over a short window (30 min) and following an overnight fast. Given that we have previously shown that obesity in humans causes muscle protein anabolic resistance only under postprandial-like conditions, and not following a fast (4), our current findings offer new insight demonstrating that the inability of 16-week high-fat high-calorie diets to blunt muscle protein synthesis also extends to exogenous leucine and nutrition *per se*.

## Conclusions

In Brown Norway rats, the impact of calorie-dense high-fat foods on energy expenditure is dependent on age, with older animals protected from the obesogenic nature of the diets. What is responsible for this discord is unclear, but if identified, could yield new approaches to avert weight gain in elderly individuals and thereby assist in the maintenance of their overall metabolic health. Notably, our findings demonstrate that the consumption of calorie-dense high-fat human foods does not induce muscle anabolic resistance to nutrition in rodents, even when examined over prolonged periods. These findings suggest that the anabolic resistance to amino acids seen in older obese adults could be due to other factors (e.g. sedentary behavior), but equally, it is permissible that current rodent models fail to fully recapitulate the consequences of obesity on muscle protein metabolism as observed in humans.

## Data Availability Statement

The raw data supporting the conclusions of this article will be made available by the authors, without undue reservation.

## Ethics Statement

The animal study was reviewed and approved by Institutional Animal Care and Use Committee of the University of Texas Medical Branch.

## Author Contributions

AE and AM designed the experiments. AE, CT, AP, VR, DA, WC, GF, NB, and AM conducted the described experiments. AE and AM analyzed collected data and wrote the initial draft of the manuscript. All authors provided critical review of the draft manuscript and gave permission to proceed with publication.

## Funding

The project was supported by a pilot grant awarded to AM by the Claude D. Pepper Older American Independence Center (NIH/NIA P30 AG024832) and the Institute of Translation Sciences at the University of Texas Medical Branch via their Clinical and Translational Science Award (NIH/NCATS UL1 TR0001439).

## Conflict of Interest

The authors declare that the research was conducted in the absence of any commercial or financial relationships that could be construed as a potential conflict of interest.

## Publisher's Note

All claims expressed in this article are solely those of the authors and do not necessarily represent those of their affiliated organizations, or those of the publisher, the editors and the reviewers. Any product that may be evaluated in this article, or claim that may be made by its manufacturer, is not guaranteed or endorsed by the publisher.
